# Suppression Trial through an Integrated Vector Management of *Aedes albopictus* (Skuse) Based on the Sterile Insect Technique in a Non-Isolated Area in Spain

**DOI:** 10.3390/insects14080688

**Published:** 2023-08-03

**Authors:** Carlos Tur, David Almenar, Mario Zacarés, Sandra Benlloch-Navarro, Ignacio Pla, Vicente Dalmau

**Affiliations:** 1 Empresa de Transformación Agraria S.A., S.M.E, M.P. (TRAGSA), Avenida de la Industria 26, 46980 Paterna, Spain; dalmenar@tragsa.es (D.A.); sbenlloc@tragsa.es (S.B.-N.); ipla@tragsa.es (I.P.); 2 Doctoral School, Universidad Católica de Valencia San Vicente Mártir, C/Guillem de Castro 94, 46001 Valencia, Spain; 3 Department of Basic and Transversal Sciences, Faculty of Veterinary and Experimental Sciences, Universidad Católica de Valencia San Vicente Mártir, C/Guillem de Castro 94, 46001 Valencia, Spain; mario.zacares@ucv.es; 4 Conselleria de Agricultura, Desarrollo Rural, Emergencia Climática y Transición Ecológica, Apdo Correos 125, 46460 Silla, Spain; dalmau_vic@gva.es

**Keywords:** SIT, vector control, mosquitoes, insect production, mass rearing, dengue, Europe

## Abstract

**Simple Summary:**

The tiger mosquito, *Aedes albopictus* (Skuse, 1894), is a competent vector of arboviruses such as dengue, Zika, and chikungunya among others. The sterile insect technique (SIT) is presented as an innovative and environmentally friendly control method to be implemented as a main component of integrated vector control management (IVM). In the Valencian region (Spain), an IVM pilot project led and funded by the Department of Agriculture, based on the use of SIT to control *Ae. albopictus*, has been carried out from 2017 to 2020. It has proven to be effective in reducing tiger mosquito populations. This manuscript analyzes the impact of the migration of wild individuals from peri-urban areas into non-isolated urban centers on the control strategy.

**Abstract:**

In recent years, *Aedes albopictus* (Skuse, 1984) has expanded its distribution globally due to its high ecological plasticity. This expansion has increased the population’s susceptibility to contracting diseases such as dengue, Zika, and chikungunya, among others, which are transmitted by this mosquito species. In the absence of effective control methods, the application of the sterile insect technique (SIT) is proposed as part of an integrated vector management (IVM) program. From 2007 to 2020, this strategy has been tested in a non-isolated mosquito population urban area of 45 ha, representative of the municipalities of the Valencian region (Spain). The population levels of adult females and eggs collected in the traps have been reduced by 70–80% compared to the control area, demonstrating its efficacy in reducing mosquito populations. This work analyzes the impact of the migration of the wild mosquito population from the peri-urban area to the urban core.

## 1. Introduction

The tiger mosquito, *Aedes albopictus* (Skuse, 1894), is a competent vector of arboviruses such as dengue, Zika, and chikungunya, along with *Aedes aegypti*. With a global distribution, its process of colonization of new areas continues to expand. In Europe alone, it has colonized more than 20 countries since 1979 [1]. Within 30 years, 68% of the continent’s area and 83% of urban regions could become suitable for *Ae. albopictus* establishment [2]. The risk associated with the spread and colonization of new areas by this species is the increase in the population at risk of contracting the diseases it transmits, as has been observed in autochthonous transmissions in recent years [3,4]. Currently, vector control stands as one of the main tools to control the transmission of these diseases, in addition to surveillance of imported cases in humans, and early detection of local transmission [5]. In Spain, a national preparedness and response plan for vector-borne diseases was published in 2016 [6] where integrated vector management (IVM) is recognized as an effective strategy to reduce transmission risk. The rapid spread of *Ae. albopictus* globally and its increasing population levels suggest that conventional methods are proving insufficient in controlling their populations, and new tools need to be incorporated. It should be noted that while vector control actions are mostly conducted in public spaces, private properties may also host numerous breeding sites. On the other hand, due to restrictions in European regulations regarding the active substances that can be used as biocides, few insecticides are available, further increasing the risk of generating resistance when used as a long-term control method [7,8,9]. However, in this context, the Sterile Insect Technique (SIT) emerges as a promising method, as it is an environmentally friendly control tool. The SIT relies on the rearing, sterilization, and release of male mosquitoes in sufficient numbers to have an impact on populations when they mate with wild females in the field, producing non-viable eggs [10]. Moreover, it does not generate resistance, and the released sterile males can disperse and access sites where virgin females are found [11]. At present, there are limited field trials of SIT application against *Ae. albopictus* as part of an IVM reported in the peer-reviewed literature, with different results, both in the reduction in eggs collected in the ovitraps and in the induced sterility rate. In pilot trials conducted in Greece and Mauritius, a significant decrease in the hatching percentage of eggs collected in ovitraps was observed. In the case of Italy, a significant reduction in the number of eggs collected was obtained when induced sterility rates reached 70–80% [12,13,14]. Furthermore, fertility rate reductions of up to 84% were achieved in 2020 with a combination of the SIT and *Bacillus thurigiensis* var. *israeliensis* (Bti) application in Germany [15]. Regarding the application of the SIT against *Ae. aegypti*, remarkable vector suppression after a 20-week sterile male release period was achieved in Cuba in 2020 [16]. One of the reported effects as a possible limiting factor in the efficacy of the SIT application, as well as other techniques such as the release of sterile male *Wolbachia*-infected mosquitoes, has been the migration of wild mosquito populations from peripheral areas into the interior of urban centers [12,13,17,18]. In light of this concern, in the Valencian region (Spain), an IVM pilot project based on the use of SIT to control *Ae. albopictus*, led and funded by the Department of Agriculture of Valencia and executed by Grupo TRAGSA, has been carried out from 2017 to 2020, conducted between March and November, to align with the seasonal population dynamics of the tiger mosquito in this region. The objective was to assess the efficacy of an IVM with the SIT as the main component for suppressing the population of *Ae. albopictus* in municipalities that were not isolated from surrounding wild populations of the same species.

## 2. Materials and Methods

### 2.1. Risk Assessment and Regulation

All activities included in this project, such as mosquito rearing, transportation, irradiation, and release have received approval from the competent regional authorities, in accordance with the national legislation for occupational risk prevention. Concerning ecological aspects, the introduction of *Ae. albopictus* in the Valencian region in recent years as the main competent vector of dengue, Zika, or chikungunya does not pose a risk of niche replacement. On the other hand, the reduced number of released females (less than 0.5% [19]) and the absence of these diseases in the Valencian region reduce considerably the risk of disease transmission in the current epidemiological situation.

### 2.2. Public Awareness

Information activities have been conducted in the municipalities involved, as well as activities in schools concerning the biology of mosquitoes, and the implementation of the SIT as a control method in an IVM. In addition, leaflets have been distributed to all households to provide information on the undertaken activities. Finally, news reports have been broadcast both in the press and on television to raise awareness about the project.

### 2.3. IVM—SIT Treated and Control Study Sites

**Figure 1:**
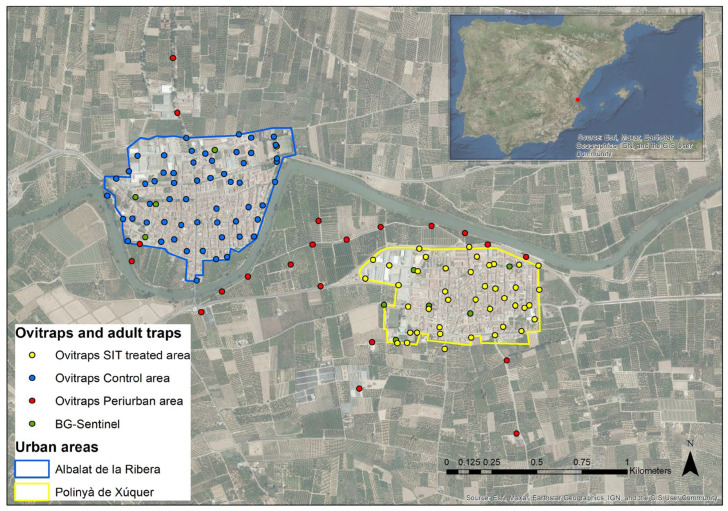
IVM-SIT and control areas. Yellow, blue, and red dots indicate the positions of the ovitraps in the SIT, control, and peri-urban areas, respectively. The green dots represent the position of the BG-Sentinel traps.

Two target areas were chosen to evaluate the efficiency of IVM based on the SIT as shown in Figure 1. The selected IVM-SIT pilot site was Polinyà de Xuquer, a municipality covering 45 hectares of urban area and 2350 inhabitants. The municipality of Albalat de la Ribera (60 ha, 4312 inhabitants), located less than one km away from the pilot area, was used as a control site. On the control site, larvicide treatments of gutters were performed by the pest control company hired by the city. In contrast, in addition to these treatments managed by the municipality, at the IVM-SIT pilot site complementary control methods, and the release of sterile males were implemented, as detailed below. Both municipalities have urban centers surrounded by citrus groves, with agriculture being the main activity in the area. Irrigated cultivation, together with the presence of scattered houses in the peripheral area, provides an ideal habitat for the establishment of *Ae. albopictus*. The climate of the region is temperate. As example, the average annual temperature in the area in 2020 was 17.8 °C, with 74.2% relative humidity [20].

### 2.4. Aedes albopictus Rearing

The colony was started in 2014 from eggs collected in ovitraps installed in different municipalities across the Valencian region. This initial colony was expanded in 2017 to produce the sterile males needed for the field trial. Adult mosquitoes were maintained in 40 × 40 × 40 cm methacrylate cages filled at a density of 10,000 individuals per cage [19]. After hatching the eggs in 1 L sealed containers with a nutrient broth solution, the larvae were reared for six days in 40 × 60 × 12 cm plastic trays filled with 5 L of deionized water, at a density of 2 larvae/mL, and fed with IAEA diet based on brewer yeast, liver powder, and tuna meal [21]. A plate separator was used for sex sorting [22], achieving an average residual percentage of females of 0.17% [19]. Plastic cups each containing approximately 750 male pupae were irradiated at 48 Gy in a Gammacell 220 irradiator. Following irradiation, each cup was introduced into 17 × 18 × 28 cm plastic containers with 2 sides of plastic mesh for adult emergence, providing access to a 10% sucrose solution. Quality control tests were applied to sterile males, including flight ability, survival, and mating competitiveness. A detailed description of the rearing and Quality Control process can be found in Tur et al. [19].

### 2.5. Aedes albopictus Transportation and Releasing

Ground releases were conducted between 3 and 6 days after irradiation, utilizing plastic containers measuring 17 × 18 × 28 cm, as described previously. Non-marked adult sterile males were transported for up to 45 min by car from the rearing facility to the pilot site. A fixed release point was defined every 100 m in the SIT pilot area (45 release points in Polinyà de Xúquer) with a uniform distribution. Based on the seasonal mosquito population dynamics observed in 2017, it was decided the following year to start the releases of sterile males in March, prior to the detection of the first eggs of the season in the ovitraps, in order to neutralize the first mosquito generations. However, due to a malfunction in the irradiator, the releases were delayed until mid-April 2018. In 2018, the sterile males were released twice per week, and 3 times a week in 2019 and 2020, usually every Monday, Wednesday, and Friday. Male sterile releases were conducted from 2018 to 2020 in Polinyà de Xúquer (see Table 1).

### 2.6. Baseline Entomological Data Collection

From 2017 to 2020, a network of ovitraps was installed in both municipalities (see Figure 1), as well as in the citrus-growing area between Polinyà de Xúquer and Albalat de la Ribera (refer to Table 2). The ovitrap consisted of a cylindrical black plastic container with a capacity of 750 mL, filled with approximately 600 mL of water. A 15 × 2.5 cm wooden stick was introduced for oviposition [23]. The wooden sticks were collected once a week, and the water in the ovitrap was completely renewed. Ovitraps were sampled every season from the last week of February to December.

The collected eggs from the ovitraps were transported in plastic containers and kept in humid conditions for three days to allow maturation. After maturation, the eggs were counted under a stereomicroscope to determine the number of hatched, unhatched, and empty eggs. After the counting process, each wooden stick with unhatched eggs was cut in 2, placed in a 90 × 14 mm petri dish sealed with parafilm along with nutrient broth and yeast hatching medium, and kept at 23–25 °C. After three days, the number of larvae in each petri dish was counted.

Concerning adult collection, 5 BG Sentinel traps were installed in each pilot site (Polinyà de Xúquer and Albalat de la Ribera) during 2019 and 2020. Adult samples were collected weekly, and the number of *Ae. albopictus* males and females were counted.

### 2.7. Measurement of Effectiveness

The following parameters have been defined to characterize mosquito populations:

Number of eggs per trap per day (*E*):

(1) $$E=\frac{\mathrm{Eggs}}{\mathrm{Traps}\times \mathrm{days}}$$

Hatch rate (*H*):

(2) $$H=\frac{\mathrm{Hatched}\;\mathrm{eggs}}{\mathrm{Eggs}}$$

Average number of females per trap per day (*F*):

(3) $$F=\frac{\mathrm{Adult}\;\mathrm{females}}{\mathrm{Traps}\times \mathrm{days}}$$

The differences between the values of these parameters in the control and the *SIT* areas provide an assessment of the impact and efficacy of the sterile male release strategy. To this end, two new variables of interest were defined and constructed based on the differences relative to the control zone:

Reduction in egg density (*D*):

(4) $$D=\frac{E_{Control}-E_{SIT}}{E_{Control}}\times 100$$

Induced sterility (*S*):

(5) $$S=\frac{H_{Control}-H_{SIT}}{H_{Control}}\times 100$$

Reduction in adult female density (*FD*):

(6) $$FD=\frac{F_{Control}-F_{SIT}}{F_{Control}}\times 100$$

### 2.8. Effect of the Agricultural Peri-Urban Zone on the SIT Pilot Area

To assess the effect of mosquito influx from the surrounding area, the parameters of interest were estimated as a function of distance to the peri-urban area. The traps were divided into four groups according to the quartiles of the distance to the peri-urban zone and were of approximately equal size. The calculations were performed using aggregated data from 2018 to 2020.

### 2.9. Additional Vector Management Control Methods in the SIT Pilot Areas

In both the SIT and control areas, the pest control company hired by the city carried out conventional control activities based on Bti and larvicide treatments in gutters. However, treatments with Bti strain AM65-52 (VectoBac^®^ 12 AS-Kenogard-Barcelona, Barcelona, Spain) were carried out in all scuppers in the SIT municipality when mosquito larvae/pupae (including other mosquito species) were detected in more than 10% of the scuppers. On the other hand, due to the high population levels in the citrus-growing area outside the urban core in 2018, it was decided to install lethal oviposition traps in the surrounding citrus area of Polinya de Xúquer, from the end of July to the end of November 2019, aiming to reduce the migration of wild population from the citrus-growing area to the urban core [24]. The lethal traps were also installed from May to November 2020. Each lethal oviposition trap consisted of a 7.5 L cylindrical black plastic container containing 5 L of water and a piece of filter paper coating the inner wall. To eliminate the larvae resulting from the eggs laid by the females, 3 mL of Bti was added to the water. The content of the traps was checked and renewed every 3 weeks. A total of 152 lethal oviposition traps were placed around the perimeter of the SIT urban nucleus, approximately 20 m apart from each other.

### 2.10. Data Analysis

A generalized linear model with an assumed negative binomial distribution was used to quantify the average number of eggs per trap per day and assess the reduction in egg density. Similarly, the hatch rate and induced sterility were estimated using a beta-binomial distribution. The goodness-of-fit and model comparisons were assessed with an analysis of deviance. Model parameters and confidence intervals were estimated by the maximum likelihood criterion and likelihood ratio tests. To mitigate week-to-week variations, a four-week aggregation of data was employed to analyze the evolution of the effectiveness parameters. The final annual assessment was carried out at the end of the year using the accumulated data for each trap. The data were analyzed with the R software version 3.6.3 [25] with ggplot2 [26] and bblme [27] packages.

## 3. Results

### 3.1. SIT Area vs. Control Area

**Figure 2:**
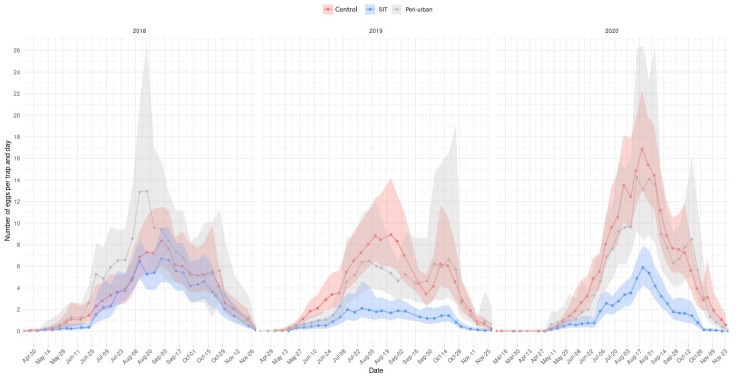
Number of eggs per trap per day in the control (red), SIT (blue), and peri-urban area (gray) from 2018 to 2020. Each marker represents the mean of eggs/trap/day of the last four weeks. The shaded areas represent the 99% confidence intervals for each zone.

During 2017, before starting the sterile male releases, no significant differences were found in the average number of eggs collected per ovitrap in the SIT and control area (Table 3, Figure 2). Sterile male releases began in April 2018 (Table 1). In 2018, although the average reduction in egg density was not significantly different from 0, for multiple weeks there was a significant reduction in oviposition. However, there was consistent induced sterility in the SIT area. As a consequence of the lower-than-expected effects on oviposition reduction, in the following two years, the first releases started in March, and the frequency and number of sterile males released increased (Table 1). The average reduction in egg density was high and significantly different from 0 in 2019 and 2020 (74.4 ± 5.3%, and 72.5 ± 4.8%, respectively). Concerning the induced sterility, results ranged from 31.7 ± 5.4% in 2019 to 19.5 ± 4.4% in 2020 (Table 3).

### 3.2. Effect of the Agricultural Peri-Urban Zone on the SIT Pilot Area

**Figure 3:**
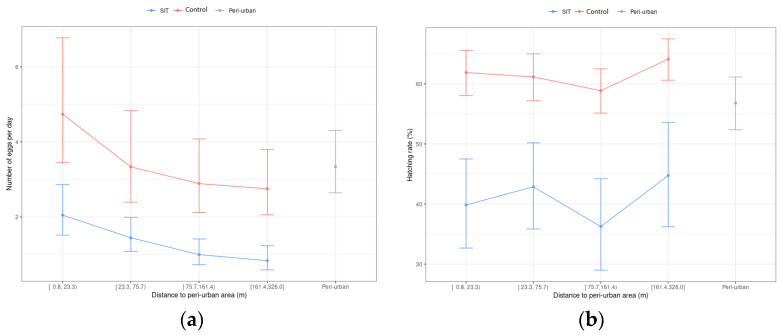
Confidence intervals (95%) of number of eggs per trap per day (**a**,**b**) hatching rate (**b**) as functions to the closest distance to the peri-urban area boundary. The limits of the groups are based on quartiles.

The average difference in the number of eggs collected per trap per day between the control zone and the peri-urban citrus area was not significantly different from 0 (in 2018, 2019 and 2020, respectively) (Table 4). After releasing sterile mosquito males in the SIT area, in 2018 there were no significant differences in the number of eggs collected in the ovitraps of the SIT and peri-urban areas (Table 4), nor in the induced sterility (C.I. 99% = [−5.4, 38.6]). However, as observed in the control area, a significant reduction in egg density was observed in 2019 (71.8 ± 6.8%) and 2020 (69.3 ± 5.8%) in the SIT treated area compared with the peri-urban citrus area, with significantly higher average egg captures in the peri-urban zone (Table 4). Significant pressure from mosquito populations of the citrus orchards surrounding the urban area was observed, with a marked gradient in the number of collected eggs per trap per day in the ovitraps, based on the distance from the perimeter of the urban center. However, this pressure did not seem to have an effect on the hatching rate (Figure 3).

## 4. Discussion

The presented pilot trial of the application of integrated vector control based on the SIT has proven to be effective in reducing *Ae. albopictus* populations in the Valencian region, Spain by up to 70–80% with respect to female density However, during the first season, only a significant reduction in hatching percentage was achieved. This could be attributed to a delayed start of sterile male releases caused by a malfunction in the irradiator. As a result, the number of males released in the first few weeks was reduced, and the releases did not commence until mid-April. In addition, in 2018, 2 releases per week were initially carried out. Based on these results, it was decided to modify the strategy for the following two seasons. Sterile male releases started one month prior to the detection of the first positive ovitraps, and the frequency of sterile male releases was increased to three releases per week. In addition, the production capacity of sterile males increased due to the introduction of improvements in rearing processes and staff training, and up to 42% more males per hectare were released compared to the first season. In relation to release frequency, studies to date have released sterile males from one to three times per week [12,13,28]. The average lifespan of sterile males in the field fluctuates greatly in temperate regions, where summer months reach high temperatures and low humidity rates. By implementing a three-day-per-week release strategy, a higher sterile-to-wild ratio can be maintained in the field while minimizing sterile male production. Considering that production costs currently exceed release costs, increasing the release frequency has emerged as an economically efficient strategy.

Concerning induced sterility on the wild population, Bellini et al. [14] suggested that when the induced sterility was below 50%, there was no impact on the reduction in adult population density. In their study, they observed that it was even necessary to induce 70–80% of sterility to have a clear effect on population reduction. In our study, although there were significant differences in hatching percentages between the control and SIT zones, the level of induced sterility was below 50%. Despite these results, a clear population reduction was observed, both in the adult populations and in the eggs collected in the ovitraps. One possible cause for the low egg sterility levels could be the influx of mated wild females from the peri-urban zone to the urban center. In many studies, *Ae albopictus* is considered to be a predominantly urban mosquito species in Europe, with a preference for highly anthropised metropolitan settings [29,30]. Nevertheless, as seen in this study, an agro-ecosystem composed mainly of fruit trees that provide a shady environment, coupled with an irrigation system that maintains high humidity and breeding sites, may represent a favorable environment for *Ae. albopictus* populations. These ecosystems also maintain human activity during the day, which may allow the females to ingest blood. However, some of these wild females reared in the agricultural areas could mate with wild males and subsequently start random dispersal, eventually reaching the outer urban areas close to the control and IVM-SIT areas. While the SIT effectively suppresses populations originating in urban areas, it has no impact on peri-urban-born females. Oliva et al. [31] observed that after a female mates with a male, there is an interval of 40 min after which, in the event of a second copulation, all the offspring produced over multiple gonotrophic cycles are the progeny of the first male. As a consequence, a female born in a peri-urban area is most likely to have copulated with a fertile male. If such a female migrates over a longer interval of time to the treated area, even if she copulates with a sterile male, her offspring will still be fully viable. This hypothesis would be consistent with the observed low rate of oviposition and capture of females in adult traps, as well as low hatching rates. In a recent review, Moore established that the average estimated distance traveled by *Ae. aegypti* mosquitoes between the release site and the recapture location was 106 m [32]. In reference to *Ae. albopictus*, the existing literature refers to a similar average dispersion, although these data are usually referred to as Mark-Release-Recapture (MRR) tests, which usually involve only laboratory-reared males [28,33]. However, studies conducted with wild populations of both males and females have reported mean mosquito travel distances exceeding 300 to 600 m [29,34]. In the case of the urban SIT area selected for this test, the average radius measured 386 m. All published trials on SIT application against *Aedes albopictus* mosquitoes have been conducted on areas smaller than 20 hectares [12,13,18]. With the exception of the project in Italy, which was carried out in an isolated area [14], the issue of wild individuals being introduced from the peripheral areas as one of the possible causes of the reduction in the impact of the technique is highlighted. To our knowledge, this is the first study on a large area to analyze the influence of wild population migration in a SIT-applied region. Isolation can be achieved by selecting an area with an ecologically inhospitable periphery for mosquitoes [30]. However, the aim of the present study was to demonstrate the effectiveness of the IVM in a representative urban area in the Valencian region, which generally presents a low level of population isolation. Another possibility is to generate artificial isolation, either by releasing sterile males in a buffer zone [35], or by employing a barrier similar to the one used in this study. Releasing sterile males into a buffer zone comes with the disadvantage of significantly increasing costs due to the need for a much larger production capacity. Although different barrier techniques have been tested [24], when releasing sterile males, it is preferable that the barrier does not affect the survival of the released males. In any case, further studies would be necessary to determine the best method of peripheral control.

In this study, larval control in scuppers or mass trapping was used as a complementary control method in the pilot areas, as the SIT is not recommended as a stand-alone control method [30]. Although the aim of the present study was to evaluate the effectiveness of an integrated control strategy primarily based on the SIT, it is important to analyze the individual impacts of each implemented control measure in the future. In any case, there may be synergistic effects as a population reduction in larval stages, for example, may increase the effectiveness of the SIT on adult populations by increasing the sterile:wild ratio. Incorporating other methods, such as door-to-door [36] or self-spreading traps [37], has shown promising results in controlling *Ae. albopictus*, thereby aiding in increasing control efficiency and reducing costs.

## 5. Conclusions

This study has demonstrated the efficacy of a SIT-based IVM as the main approach for the control of *Ae. albopictus* in a non-isolated environment. At the same time, it helps to assess the limits of efficacy in a more realistic environment in many regions. To further mitigate *Ae. albopictus* population levels, it would be crucial to evaluate complementary and efficient population control techniques and barriers.

## Figures and Tables

**Table 1:** Descriptive data on the sterile male releases in Polinyà de Xúquer.

Season	Releases Start Date	Releases End Date	n Releases/ Week	n Releases	Total n Sterile Males Released	Average Sterile Males Released/Week/ha
2018	17 April 2018	5 December 2018	2	68	2,155,800	1409
2019	4 March 2019	29 November 2019	3	109	3,034,250	1729
2020	2 March 2020	30 November 2020	3	127	4,628,250	2446

**Table 2:** Traps installed in the pilot areas.

Pilot Site	n Ovitraps	n BG Sentinel Traps
Polinyà de Xúquer	45	5
Albalat de la Ribera	58	5
Periferial area	22	-

**Table 3:** Mean number of eggs per trap per day (E), hatch rate (H), average number of adult females per trap per day (F), reduction in egg density (D), induced sterility (S), and reduction in adult female density (FD), in the control and SIT area per season.

Season	Site	E		H		F		D	S	FD
		Estimate ± SE	Difference between Sites CI (99%)	Estimate ± SE	Difference between Sites CI (99%)	Estimate ± SE	Difference between Sites CI (99%)	Estimate ± SE CI (99%)	Estimate ± SE CI (99%)	Estimate ± SE CI (99%)
2017	Control	4.3 ± 0.5	[−0.1, 3.2]							
	SIT	2.9 ± 0.4								
2018	Control	2.6 ± 0.3	[−0.5, 1.6]	51.2 ± 1.9	[7.1, 23.6]			21.3 ± 12.7	30.4 ± 5.6	
	SIT	2.1 ± 0.3		35.6 ± 2.5				[−20.8, 48.3]	[14.6, 43.9]	
2019	Control	3 ± 0.4	[1.3, 3.4]	65 ± 2	[10.5, 30.3]	1.0 ± 0.3	[0.1, 1.6]	74.4 ± 5.3	31.7 ± 5.4	80.5 ± 9.8
	SIT	0.8 ± 0.1		44.4 ± 3.2		0.2± 0.1		[55, 82.9]	[16.9, 45.2]	[39.2, 92.7]
2020	Control	4.6 ± 0.4	[2.2, 4.7]	63.1 ± 1.4	[4.7, 20]	2.4 ± 0.4	[0.7–2.9]	72.5 ± 4.8	19.5 ± 4.4	71.3 ± 8.4
	SIT	1.3 ± 0.2		50.8 ± 2.5		0.7± 0.2		[55.6, 82.4]	[7.8, 30.9]	[45.0, 88.7]

**Table 4:** Confidence interval at the 99% level for the absolute difference in the average eggs captured in ovitraps between the peri-urban zone and each of the 2 study areas (SIT and Control), across the years.

*Year*	*Peri-Urban—CONTROL*	*Peri-Urban—SIT*
***2018***	[−32.8, 60.5]	[−5.4, 38.6]
***2019***	[−91.4, 39.7]	[46.6, 85.3]
***2020***	[−70.6, 28.6]	[48.7, 81.3]

## Data Availability

Not applicable.
